# Supplementary material for: Identification of Retrocopies in Lepidoptera and Impact on Domestication of Silkworm
Source: Genes (Basel). 2024 Dec 21;15(12):1641. doi: 10.3390/genes15121641 (PMC11675541; doi:10.3390/genes15121641)
Supplement: Supplementary file 1 [file genes-15-01641-s001.zip › genes-3319768-supplementary.pdf]

Supplementary Table S1. Sources of genomes in lepidopteran insect.

Species	NCBI No
B. anynana	GCF_900239965.1
B. mandarina	GCF_003987935.1
M. sexta	GCF_000262585.1
P. xylostella	GCF_000330985.1
S. frugiperda	GCF_011064685.1
S. litura	GCF_002706865.1
T. ni	GCF_003590095.1

Supplementary Table S2 Information of genomes in lepidopteran insect

Name	Genome (Mb)	Number of genes	Number of protein
B. anynana	475.4	15654	21603
B. mandarina	398.6	13731	19224
B. mori	445.1	20313	20313
M. sexta	419.4	16654	21890
P. xylostella	393.5	19211	21674
S. frugiperda	486.3	20644	30907
S. litura	438.9	17215	24319
T. ni	368.2	16952	23658

Supplementary Table S3 Information on transcriptome data of silkworm

Name	Tissue	Stage	Name	Tissue	Stage
CJ_A_1	Antenna	moth-day-1	BPP1_2	Epidermis	pupa-day-1
CJ_A_2	Antenna	moth-day-1	BPP1_3	Epidermis	pupa-day-1
CJ_A_3	Antenna	moth-day-1	BPPP_1	Epidermis	pre-pupa
QS_4M_1	ASG	L4-molting	BPPP_2	Epidermis	pre-pupa
QS_4M_2	ASG	L4-molting	BPPP_3	Epidermis	pre-pupa
QS_4M_3	ASG	L4-molting	BPW_1	Epidermis	wandering
QS_5Q_2	ASG	L5D0	BPW_2	Epidermis	wandering
QS_5Q_3	ASG	L5D0	BPW_3	Epidermis	wandering
QS_L4D3_1	ASG	L4D3	ZFT_4M_1	Fatbody	L4-molting
QS_L4D3_2	ASG	L4D3	ZFT_4M_2	Fatbody	L4-molting
QSL5D3Q_1	ASG	L5D3	ZFT_4M_3	Fatbody	L4-molting
QSL5D3Q_2	ASG	L5D3	ZFT_5Q_1	Fatbody	L5D0
QSL5D3Q_3	ASG	L5D3	ZFT_5Q_2	Fatbody	L5D0
QSPP_1	ASG	pre-pupa	ZFT_5Q_3	Fatbody	L5D0
QSPP_2	ASG	pre-pupa	ZFTA_1	Fatbody	moth-day-1
QSPP_3	ASG	pre-pupa	ZFTA_2	Fatbody	moth-day-1
QSW_1	ASG	wandering	ZFTA_3	Fatbody	moth-day-1
QSW_2	ASG	wandering	ZFT_L4D3_1	Fatbody	L4D3
QSW_3	ASG	wandering	ZFT_L4D3_2	Fatbody	L4D3
BP_4M_1	Epidermis	L4-molting	ZFT_L4D3_3	Fatbody	L4D3
BP_4M_2	Epidermis	L4-molting	ZFTL5D3Q_1	Fatbody	L5D3
BP_4M_3	Epidermis	L4-molting	ZFTL5D3Q_2	Fatbody	L5D3
BP_5Q_1	Epidermis	L5D0	ZFTL5D3Q_3	Fatbody	L5D3
BP_5Q_2	Epidermis	L5D0	ZFTP1_2	Fatbody	pupa-day-1
BP_5Q_3	Epidermis	L5D0	ZFTP1_3	Fatbody	pupa-day-1
BP_L4D3_1	Epidermis	L4D3	ZFT_p4_1	Fatbody	pupa-day-4
BP_L4D3_2	Epidermis	L4D3	ZFT_p4_2	Fatbody	pupa-day-4
BP_L4D3_3	Epidermis	L4D3	ZFT_p4_3	Fatbody	pupa-day-4
BPL5D3Q_1	Epidermis	L5D3	ZFT_p7_1	Fatbody	pupa-day-7-8
BPL5D3Q_2	Epidermis	L5D3	ZFT_p7_2	Fatbody	pupa-day-7-8
BPL5D3Q_3	Epidermis	L5D3	ZFT_p7_3	Fatbody	pupa-day-7-8
BPP1_1	Epidermis	pupa-day-1	ZFTPP_1	Fatbody	pre-pupa

Name	Tissue	Stage	Name	Tissue	Stage
ZFTPP_2	Fatbody	pre-pupa	L5D3-1XY	Hemolymph	L5D3
ZFTPP_3	Fatbody	pre-pupa	L5D3-2XY	Hemolymph	L5D3
ZFTW_1	Fatbody	wandering	L5D3-3XY	Hemolymph	L5D3
ZFTW_2	Fatbody	wandering	XY_ 5Q-1	Hemolymph	L5D0
ZFTW_3	Fatbody	wandering	XY_ 5Q-2	Hemolymph	L5D0
ZFTP1_1	Fatbody	pupa-day-1	XY_ 5Q-3	Hemolymph	L5D0
HW_1	Head	wandering	XYW_1	Hemolymph	wandering
HW_2	Head	wandering	XYW_2	Hemolymph	wandering
HW_3	Head	wandering	XYW_3	Hemolymph	wandering
head_4LD3_1	Head	L4D3	foot_A_1	Leg	moth-day-1
head_4LD3_2	Head	L4D3	foot_A_2	Leg	moth-day-1
head_4LD3_3	Head	L4D3	foot_A_3	Leg	moth-day-1
head_4M_1	Head	L4-molting	MSG_4M_2	Ma-Tubule	L4-molting
head_4M_2	Head	L4-molting	MSG_4M_3	Ma-Tubule	L4-molting
head_4M_3	Head	L4-molting	MSG_5Q_1	Ma-Tubule	L5D0
head_5Q_1	Head	L5D0	MSG_5Q_3	Ma-Tubule	L5D0
head_5Q_2	Head	L5D0	MSG_L4D3_1	Ma-Tubule	L4D3
head_5Q_3	Head	L5D0	MSG_L4D3_2	Ma-Tubule	L4D3
head_A_1	Head	moth-day-1	MSG_L4D3_3	Ma-Tubule	L4D3
head_A_2	Head	moth-day-1	MSGL5D3Q_1	Ma-Tubule	L5D3
head_A_3	Head	moth-day-1	MSGL5D3Q_2	Ma-Tubule	L5D3
HL5D3Q_1	Head	L5D3	MSGL5D3Q_3	Ma-Tubule	L5D3
HL5D3Q_2	Head	L5D3	MSGPP_1	Ma-Tubule	pre-pupa
HL5D3Q_3	Head	L5D3	MSGPP_2	Ma-Tubule	pre-pupa
HPP_1	Head	pre-pupa	MSGPP_3	Ma-Tubule	pre-pupa
HPP_2	Head	pre-pupa	MSGW_1	Ma-Tubule	wandering
HPP_3	Head	pre-pupa	MSGW_2	Ma-Tubule	wandering
4D3_MIX1	Hemolymph	L4D3	MSGW_3	Ma-Tubule	wandering
4D3_MIX2	Hemolymph	L4D3	MSG_4M_1	Ma-Tubule	L4-molting
4D3_MIX3	Hemolymph	L4D3	ZS_4M_1	MSG	L4-molting
4M_MIX1	Hemolymph	L4-molting	ZS_4M_2	MSG	L4-molting
4M_MIX2	Hemolymph	L4-molting	ZS_4M_3	MSG	L4-molting
4M_MIX3	Hemolymph	L4-molting	ZS_5Q_1	MSG	L5D0

Name	Tissue	Stage	Name	Tissue	Stage
ZS_5Q_2	MSG	L5D0	ZC_4M_2	Midgut	L4-molting
ZS_5Q_3	MSG	L5D0	ZC_4M_3	Midgut	L4-molting
ZS_L4D3_1	MSG	L4D3	LC_4M_1	Ovary	L4-molting
ZS_L4D3_2	MSG	L4D3	LC_4M_2	Ovary	L4-molting
ZS_L4D3_3	MSG	L4D3	LC_4M_3	Ovary	L4-molting
ZSL5D3Q_1	MSG	L5D3	LC_5Q_1	Ovary	L5D0
ZSL5D3Q_2	MSG	L5D3	LC_5Q_2	Ovary	L5D0
ZSL5D3Q_3	MSG	L5D3	LC_5Q_3	Ovary	L5D0
ZSPP_1	MSG	pre-pupa	LC_L4D3_1	Ovary	L4D3
ZSPP_2	MSG	pre-pupa	LC_L4D3_2	Ovary	L4D3
ZSPP_3	MSG	pre-pupa	LC_L4D3_3	Ovary	L4D3
ZSW_1	MSG	wandering	LCL5D3Q_1	Ovary	L5D3
ZSW_2	MSG	wandering	LCL5D3Q_3	Ovary	L5D3
ZSW_3	MSG	wandering	LCP1_1	Ovary	pupa-day-1
ZC_5Q_1	Midgut	L5D0	LCP1_2	Ovary	pupa-day-1
ZC_5Q_2	Midgut	L5D0	LCP1_3	Ovary	pupa-day-1
ZC_5Q_3	Midgut	L5D0	LCPP_1	Ovary	pre-pupa
ZC_L4D3_1	Midgut	L4D3	LCPP_2	Ovary	pre-pupa
ZC_L4D3_2	Midgut	L4D3	LCPP_3	Ovary	pre-pupa
ZC_L4D3_3	Midgut	L4D3	LCW_1	Ovary	wandering
ZCL5D3Q_1	Midgut	L5D3	LCW_2	Ovary	wandering
ZCL5D3Q_2	Midgut	L5D3	LCW_3	Ovary	wandering
ZCL5D3Q_3	Midgut	L5D3	HS_L4D3_3	PSG	L4D3
ZCP1_1	Midgut	pupa-day-1	HSL5D3Q_1	PSG	L5D3
ZCP1_2	Midgut	pupa-day-1	HSL5D3Q_2	PSG	L5D3
ZCP1_3	Midgut	pupa-day-1	HSL5D3Q_3	PSG	L5D3
ZCPP_1	Midgut	pre-pupa	HSPP_1	PSG	pre-pupa
ZCPP_2	Midgut	pre-pupa	HSPP_2	PSG	pre-pupa
ZCPP_3	Midgut	pre-pupa	HSPP_3	PSG	pre-pupa
ZCW_1	Midgut	wandering	HSW_1	PSG	wandering
ZCW_2	Midgut	wandering	HSW_2	PSG	wandering
ZCW_3	Midgut	wandering	HSW_3	PSG	wandering
ZC_4M_1	Midgut	L4-molting	HS_4M_1	PSG	L4-molting

Name	Tissue	Stage	Name	Tissue	Stage
HS_4M_2	PSG	L4-molting	JCPP_2	Testis	pre-pupa
HS_4M_3	PSG	L4-molting	JCPP_3	Testis	pre-pupa
HS_5Q_2	PSG	L5D0	JCW_1	Testis	wandering
HS_5Q_3	PSG	L5D0	JCW_2	Testis	wandering
HS_L4D3_1	PSG	L4D3	JCW_3	Testis	wandering
HS_L4D3_2	PSG	L4D3	chest_A1	Thorax	moth-day-1
JC_4M_1	Testis	L4-molting	chest_A2	Thorax	moth-day-1
JC_4M_1	Testis	L4-molting	chest_A3	Thorax	moth-day-1
JC_4M_1	Testis	L4-molting	QG1	Trachea	pre-pupa
JC_5Q_1	Testis	L5D0	QG2	Trachea	pre-pupa
JC_5Q_2	Testis	L5D0	QG3	Trachea	pre-pupa
JC_5Q_3	Testis	L5D0	QG_4LD3_1	Trachea	L4D3
JCA_1	Testis	moth-day-1	QG_4LD3_2	Trachea	L4D3
JCA_2	Testis	moth-day-1	QG_4LD3_3	Trachea	L4D3
JCA_3	Testis	moth-day-1	QG_4M_1	Trachea	L4-molting
JC_L4D3_1	Testis	L4D3	QG_4M_2	Trachea	L4-molting
JC_L4D3_2	Testis	L4D3	QG_4M_3	Trachea	L4-molting
JC_L4D3_3	Testis	L4D3	QG_5Q_1	Trachea	L5D0
JCL5D3Q_1	Testis	L5D3	QG_5Q_2	Trachea	L5D0
JCL5D3Q_2	Testis	L5D3	QGL5D3Q_1	Trachea	L5D3
JCL5D3Q_3	Testis	L5D3	QGL5D3Q_2	Trachea	L5D3
JC_P1_1	Testis	pupa-day-1	QGL5D3Q_3	Trachea	L5D3
JC_p4_1	Testis	pupa-day-4	QGWQ_1	Trachea	wandering
JC_p4_2	Testis	pupa-day-4	QGWQ_2	Trachea	wandering
JC_p4_3	Testis	pupa-day-4	QGWQ_3	Trachea	wandering
JC_p7_1	Testis	pupa-day-7-8	wing_A_1	Wing	moth-day-1
JC_p7_2	Testis	pupa-day-7-8	wing_A_2	Wing	moth-day-1
JC_p7_3	Testis	pupa-day-7-8	wing_A_3	Wing	moth-day-1
JCPP_1	Testis	pre-pupa			

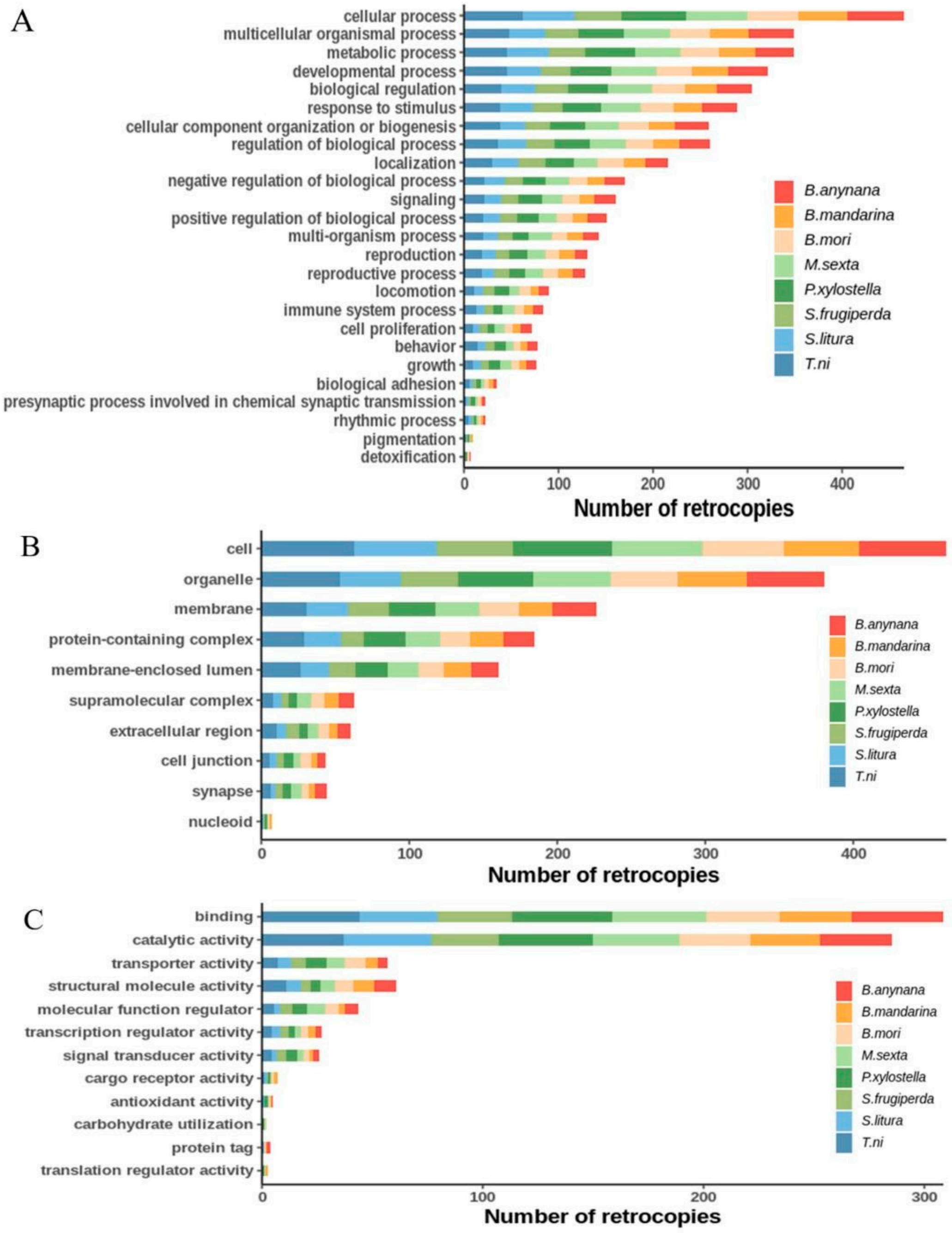

Supplementary Figure S1. GO terms of retrocopies in the lepidopteran insect genomes

(A) Biological process. (B) Cellular component. (C) Molecular function.

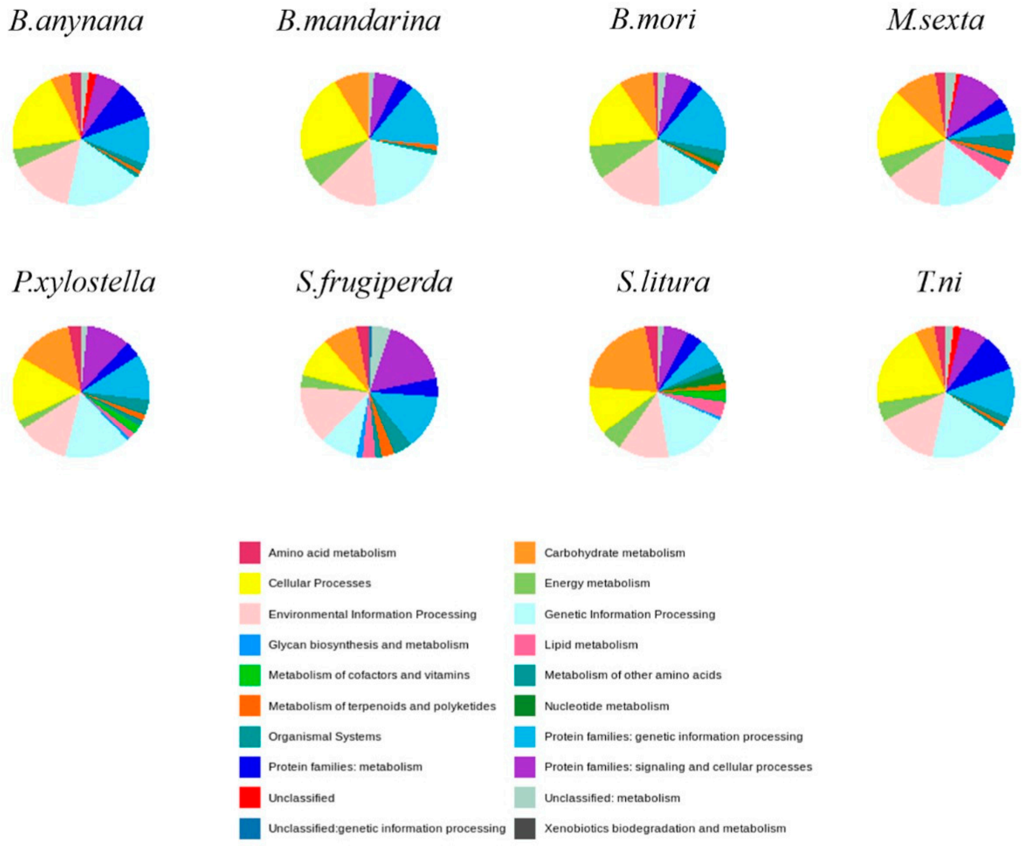

Supplementary Figure S2. KEGG pathway of retrocopies in the lepidopteran insect genomes
